# *In vitro* Activity and Heteroresistance of Omadacycline Against Clinical *Staphylococcus aureus* Isolates From China Reveal the Impact of Omadacycline Susceptibility by Branched-Chain Amino Acid Transport System II Carrier Protein, Na/Pi Cotransporter Family Protein, and Fibronectin-Binding Protein

**DOI:** 10.3389/fmicb.2019.02546

**Published:** 2019-11-08

**Authors:** Bing Bai, Zhiwei Lin, Zhangya Pu, Guangjian Xu, Fan Zhang, Zhong Chen, Xiang Sun, Jinxin Zheng, Peiyu Li, Qiwen Deng, Zhijian Yu

**Affiliations:** ^1^Department of Infectious Diseases and Shenzhen Key Lab of Endogenous Infections, Shenzhen Nanshan People’s Hospital and the 6th Affiliated Hospital of Shenzhen University Health Science Center, Shenzhen, China; ^2^Quality Control Center of Hospital Infection Management of Shenzhen, Shenzhen Nanshan People’s Hospital, Guangdong Medical University, Shenzhen, China; ^3^Key Laboratory of Viral Hepatitis of Hunan Province, Department of Infectious Diseases, Xiangya Hospital, Central South University, Changsha, China

**Keywords:** omadacycline, *Staphylococcus aureus*, antimicrobial activity, multilocus sequence typing, tetracycline specific resistance genes

## Abstract

Omadacycline (Omad), a new tetracycline (Tet)-class broad-spectrum aminomethylcycline, has been reported to exhibit excellent potency against Gram-positive bacteria, including *Staphylococcus aureus* and Enterococci. The aim of this study was to evaluate the *in vitro* activity and heteroresistance characteristics of Omad in clinical *S. aureus* isolates from China and investigate Omad resistance mechanisms. A sample of 263 non-duplicate clinical *S. aureus* isolates [127 methicillin-resistant (MRSA) and 136 methicillin-sensitive (MSSA)] were collected retrospectively. Our data indicated that Omad exhibited excellent *in vitro* activity against both MRSA and MSSA. Omad heteroresistance frequencies were 3.17% (4/126) in MRSA and 12.78% (17/133) in MSSA. No mutations in Tet target sites, (five 16SrRNA copies and 30S ribosomal protein S10) were present in heteroresistance-derived clones, whereas Tet target site mutations contribute to induced Omad resistance in *S. aureus in vitro*. RNA sequencing (RNA-Seq) revealed that overexpression of branched-chain amino acid transport system II carrier protein and Na/Pi cotransporter family protein contributes to Omad heteroresistance emergence. Whole-genome sequencing demonstrated that the genetic mutation of fibronectin-binding protein (FnBP) could increase the Omad MIC. In conclusion, Omad heteroresistance risk should be considered in clinical isolates with MICs ≥ 0.5 mg/L and Omad susceptibility in *S. aureus* may be affected by efflux pump proteins (i.e., a branched-chain amino acid transport system II carrier protein and an Na/Pi cotransporter family protein), and FnBP.

## Introduction

*Staphylococcus aureus* is a pervasive human pathogen that causes infectious diseases ranging in severity from superficial skin abscesses to bacteremia and septic shock (Calfee, 2017). Although the incidence of methicillin-resistant *S. aureus* (MRSA) infection appears to be declining worldwide, the incidence of bacteremia and severe community-acquired infection caused by methicillin-susceptible *S. aureus* (MSSA) continues to be an important human health threat (Bal et al., 2017). Both MRSA and MSSA infections remain a major clinical problem exacerbated by the ongoing evolution and transmission of traits engendering resistance or reduced susceptibility to current last-line antimicrobial agents, including linezolid, daptomycin, tigecycline (Tig), and vancomycin. Thus, there remains an urgent need for the development of new antimicrobial agents (Bal et al., 2017; Calfee, 2017).

Omadacycline [7-dimethylamino, 9-(2,2-dimethyl-propyl)-aminomethylcycline; Omad] is a recently developed semisynthetic aminomethylcycline belonging to the tetracycline (Tet) family (Pfaller et al., 2017a). It has extraordinarily broad-spectrum antimicrobial activity against Gram-positive and -negative bacteria, including difficult-to-treat multidrug resistant bacteria, such as MRSA and vancomycin-resistant *enterococci* (Pfaller et al., 2017a). Like other Tet drugs, Omad is a potent inhibitor of the bacterial ribosome that inhibits bacterial protein synthesis by binding 30S ribosomal subunits during translation (Draper et al., 2014; Honeyman et al., 2015; Heidrich et al., 2016; Villano et al., 2016). Omad has the advantage of being minimally affected by classical Tet resistance mechanisms, including efflux pumps and ribosomal protection mechanisms. Omad also exhibits lower minimum inhibitory concentration (MIC) values against multidrug resistant bacteria than minocycline and Tig (Draper et al., 2014; Honeyman et al., 2015; Heidrich et al., 2016; Villano et al., 2016), making it a novel potential last-resort antibiotic for difficult-to-treat bacteria infections (Noel et al., 2012; Macone et al., 2014; Pfaller et al., 2017b; Zhang et al., 2018).

Heteroresistance means that there are population-wide variable responses to antibiotics. Several reports, including the earliest studies describing the phenomenon, applied this definition without specifying a particular antibiotic concentration range (El-Halfawy and Valvano, 2013, 2015). Previously, we obtained MICs and heteroresistance occurrence data for the new generation Tet-class drug erevacycline in clinical *S. aureus* isolates from China, underscoring the importance and necessity of investigating the characteristics of new-generation Tet derivatives (Zhang et al., 2018; Zheng et al., 2018). There are limited data regarding Omad activity against clinical *S. aureus* isolates from China. Heteroresistance development in last-resort antibiotics can hinder efficacy and, ultimately, lead to treatment failure (Claeys et al., 2016; Zhang et al., 2018; Zheng et al., 2018). Thus, it is important to establish potential factors associated with Omad heteroresistance development.

Reduced susceptibility to Tig, an archetype new-generation Tet-class drug, in several species of bacteria has been associated with genetic mutations affecting 30S ribosomal subunits, including altered copy numbers of genes encoding 16S rRNA and 30S ribosomal proteins S3 and S10 (Nguyen et al., 2014; Lupien et al., 2015; Grossman, 2016; Argudin et al., 2018). Tig resistance has been related to regulators of cell envelop proteins, including efflux pumps (e.g., SoxS, MarA, RamA, and Rob) in Gram-negative enterobacteria and MepR/MepA in *S. aureus* (Nguyen et al., 2014; Grossman, 2016; Linkevicius et al., 2016; Dabul et al., 2018). The possible influences of 30S ribosomal subunit mutations and the overexpression of efflux proteins on Omad heteroresistance and resistance in *S. aureus* has not been resolved and needs to be further studied.

The main purpose of this study was to investigate the *in vitro* antimicrobial activity of Omad and to use population analysis profile (PAP) analysis to evaluate the occurrence of Omad heteroresistance in *S. aureus* isolates from China. We examined Omad heteroresistance mechanisms in *S. aureus* by conducting polymerase chain reaction (PCR) experiments to detect genetic mutations in 30S ribosome units, administering efflux protein inhibitors (Zhang et al., 2018; Zheng et al., 2018), and conducting RNA sequencing (RNA-Seq) studies. Furthermore, we used *in vitro* induction of resistance under Omad pressure and next generation sequencing (NGS) to compare Omad-sensitive and -resistant isolates and uncover molecular factors involved in Omad resistance.

## Materials and Methods

### Bacterial Isolates

A total of 263 non-duplicate clinical *S. aureus* isolates (127 MRSA and 136 MSSA) were collected from Shenzhen Nanshan People’s Hospital, a tertiary hospital with 1,200 beds in China, between 2008 and 2016. The specimen sources are summarized in Supplementary Figure S1. *S. aureus* ATCC29213 was used as a quality control organism. All procedures involving human participants were performed in accordance with the ethical standards of Shenzhen University and the 1964 Helsinki declaration and its later amendments, or comparable ethical standards. For this type of study, formal consent is not required.

### Antimicrobial Susceptibility

*Staphylococcus aureus* antimicrobial susceptibilities to a panel of antibiotics (i.e., amikacin, erythromycin, ciprofloxacin, rifampicin, Tet, tobramycin, vancomycin, linezolid, nitrofurantoin, amoxicillin/clavulanate, and quinupristin) with the VITEK 2 system (BioMérieux, Marcy l’Etoile, France) and susceptibility breakpoints based on CLSI guidelines (2016). Omad was obtained from The Medicines Company (Med Chem Express, Monmouth Junction, NJ, United States). Omad MICs were determined with the agar dilution method according to CLSI guidelines (Klionsky et al., 2016). We employed three Omad MIC levels (≤0.25 mg/L, 0.5 mg/L, and ≥1 mg/L) in our antimicrobial susceptibility analysis. The following Acute Bacterial Skin and Skin Structure Infections Omad susceptibility breakpoints recommended by FDA criteria were adopted: ≤0.5 mg/L for susceptibility, 1 mg/L for intermediate status, and ≥2 mg/L for resistance.

### PAP Development

Omad heteroresistance in *S. aureus* was determined by PAP development as described previously (Zhang et al., 2018; Zheng et al., 2018) with a MIC cut-off criterion of ≤0.5 mg/L. Briefly, 50-μL aliquots (108 bacterial colony forming units) were spread onto Müller-Hinton broth plates containing serial dilutions of Omad (in mg/L): 0.5, 1, 2, and 3. Colonies were counted on Omad-containing plates after 24 h of incubation at 37°C. According to the criteria described above, we defined 2 mg/L as the susceptibility breakpoint for PAP determination of *S aureus*. For Omad-resistant subpopulations detected among Omad-susceptible *S. aureus* isolate colonies grown on agar plates with 2 mg/L Omad with a detection limit of ≥5 colony forming units/ml, the parental isolates were considered to have Omad heteroresistance. Two heteroresistance-derived colonies were selected randomly from plates and their Omad and Tig MICs were measured by agar dilution according to CLSI guidelines and then subjected to PCRs, efflux inhibition, and transcriptional analysis (Zhang et al., 2018).

### Polymerase Chain Reaction

Genomic DNA was extracted from isolates with Lysis Buffer for Microorganisms to Direct PCR (Takara Bio Inc., Japan). Tet resistance genes encoding Tet(K), Tet(L), Tet(M), and Tet(O) were detected by PCR analysis as described previously (Bai et al., 2018). The presence of 30S ribosomal subunit mutations, including five separate copies of the 16S rRNA gene, the genes encoding the 30S ribosomal proteins S3 and S10, and the genes encoding recombinase (RecB) and fibronectin-binding protein (FnBP) were analyzed by PCR and sequence alignment (primer sequences listed in Supplementary Table S1). Multi-locus sequence typing (MLST) was conducted to identify the distributions of sequence types (STs) among MRSA and MSSA isolates. PCR conditions recommended for locus amplification^1^ were employed.

### Efflux Inhibition

The role of efflux pumps in Omad heteroresistance was evaluated with the efflux pump inhibitors phenylalanine-arginine-β-naphthylamide (PaβN) and carbonyl cyanidem-chlorophenylhydrazine (CCCP; both from Sigma). Omad MICs were determined by the agar dilution method in the presence and absence of 50 mg/L PAβN or 16 mg/L CCCP. Inhibition was confirmed based on a ≥4-fold MIC reduction (Osei Sekyere and Amoako, 2017; Zhang et al., 2018).

### *In vitro* Induction of Omad-Resistance Under Omad Pressure

Seven parental *S. aureus* isolates, including six clinical isolates (MSSAs: CHS221, CHS165, and 149. MRSAs: CHS759, CHS810, and CHS820) and a well-characterized antibiotic-susceptible MS4 strain, were used to select Omad-resistant isolates. These isolates were subcultured serially in Mueller-Hinton broth containing gradual increasing Omad concentrations with the initial concentration being MIC equivalents followed by successive increases to 2×, 4×, 8×, and 16× MICs (Yao et al., 2018), with four passages at each concentration. Isolates from the passages of each concentration were stored at −80°C in Mueller-Hinton broth containing 40% glycerol for subsequent Tet-target site genetic mutation detection, subsequent MIC assays, next generation sequencing, and PCR analysis. Killing curves were performed on the CHS221 (wild-type MSSA strain), CHS221-O (Omad heteroresistance MSSA strain), CHS221-1Δ: (Omad resistance MSSA strain), CHS759 (wild-type MRSA strain), CHS759-O (Omad heteroresistance MRSA strain), and CHS759-1Δ: (Omad resistance MRSA strain). Tubes containing Omad at concentrations corresponding to 0 and 4 mg/L were inoculated with a suspension of each test strain, yielding to a final bacterial density of 8 × 106 cfu/ml. The killing curves shown Omad-resistant strain could grow well at concentrations corresponding to 4 mg/L (Supplementary Figure S2).

### RNA-Seq

Wild-type strain CHS221 (S221) and its heteroresistance-derived *S. aureus* isolate CHS221-O (S221-O1) were grown and prepared for total RNA extraction with TRIzol reagent (Invitrogen, Carlsbad, CA, United States) as described previously (Zheng et al., 2018). RNA-Seq of the aforementioned parental and heteroresistance-derived isolates was performed as reported previously (Lin et al., 2017). Raw data (raw reads) of fastq format were firstly processed through in-house perl scripts. In this step, clean data (clean reads) were obtained by removing reads containing adapter, reads containing ploy-N and low quality reads from raw data. All the downstream analyses were based on the clean data with high quality. The raw data from the samples were analyzed in Subread software; raw counts for each group were normalized and processed in the EdgeR Bioconductor software package. 1.3-fold differences in expression level by RNA-Sequencing were considered to be differentially expressed genes (DEGs). The RNA sequencing outcomes for two strains were deposited in the NCBI database (BioProject accession number PRJNA505108).

### Quantitative Real Time (qRT)-PCR Analysis

We selected 30 DEGs based on our RNA-Seq results and employed qRT-PCR to compare transcriptional expression levels between the parental and heteroresistance-derived strains as described in detail previously (Zheng et al., 2018). The transcriptional expression levels of the eight candidates genes (USA300HOU_RS00705, USA300 HOU_RS03535, USA300 HOU_RS01625, USA300 HOU_RS00550, USA300HOU_ RS13205, USA300HOU_RS13945, USA300HOU_RS10505, and USA300HOU_RS00660) were further analyzed and compared among the CHS165 (MSSA), 149 (MSSA), CHS759 (MRSA), CHS810 (MRSA), and CHS820 (MRSA) parental strains and their derivative heteroresistant and resistant strains. Total RNA of bacteria was extracted using the RNeasyH Mini Kit (QIAGEN, Hilden, Germany) following the manufacturer’s instructions. The extracted RNA was reverse transcribed into cDNA using iScript reverse transcriptase (Bio-Rad, Hercules, CA, United States) with incubation for 5 min at 25°C, followed by 30 min at 42°C and 5 min at 85°C. Subsequently, qRT-PCRs were performed using SYBR green PCR reagents (Premix EX TaqTM, Takara Biotechnology, Dalian, China) in the Mastercycler realplex system (Eppendorf AG, Hamburg, Germany) with amplification conditions of 95°C for 30 s, 40 cycles of 95°C for 5 s and 60°C for 34 s, followed by melting curve analysis. The control gene *gyrB* was used to normalize gene expression. Threshold cycle (Ct) numbers were determined by detection system software and analyzed with the 2^–44^Ct method and three replicates have been made. The qRT-PCR primers used are listed in Supplementary Table S2.

### Next Generation Sequencing

An Omad resistant *S. aureus* strain, MS4O8, was derived from an Omad-susceptible isolate, MS4. Chromosomal DNA was extracted from MS4O8 cells for NGS. Nextera shotgun libraries and whole genome sequencing were performed by Novogene Company (Beijing, China). Illumina PE150 sequencing data were mapped against the CP009828 *S. aureus* MS4 strain reference genome in bwa mem software (v0.7.5a)^2^ with standard parameters. Small nucleotide polymorphisms and small insertions/deletions were detected in MS408 cells, relative to MS4, in MUMmer (version 3.23). A custom script was used to detect substitutions, insertions, and deletions that might be impacting protein coding regions. Binary alignment/map files of the sequenced strains were deposited in the NCBI database (BioProject accession number PRJNA511962).

### Gene Overexpression

Full-length candidate genes, including USA300HOU_RS00550 (encodes a Na/Pi cotransporter family protein), USA300HOU_ RS01625 (encodes a branched-chain amino acid transport system II carrier protein), USA300HOU_RS03535, USA300HOU_ Tet(K), NI36_12460 (FnBP), and NI36_00170 (RecB), were amplified with extra double enzyme sites from total DNA extracted from USA300HOU and MS4 isolates. RecB-M is RecB with a mutation (R10R, I23V, I23N, H24N, H24Q, V29L, and V35M) and FnBP-M is FnBP with a mutation (T672S and I665V). RecB-M and FnBP-M DNA fragments were amplified from MS4O8 by PCR. The candidate gene fragments were each integrated into separate pIB166 vectors, and their encoded target protein were induced with 2 mM chromium chloride (Wu et al., 2012). The primers used for vector constructs in this study are listed in Supplementary Table S3. Positively vector transformed *S. aureus* clones were selected with chloramphenicol and verified by PCR and Sanger sequencing. The overexpression plasmids were transformed separately into three to five Omad-sensitive isolates and their integrations was confirmed by PCR and Sanger sequencing. Candidate gene transcriptional levels were measured by qRT-PCR, as described above. Omad and Tig MICs for these derivatives were determined and heteroresistance occurrence in these derivatives was tested by PAP analysis under Omad pressure as described above.

### Statistical Analysis

Continuous data were analyzed with Student’s *t*-tests and one-way factorial analyses of variance (ANOVAs) in SPSS software package (version 17.0, Chicago, IL, United States). *P*-values < 0.05 were regarded as statistically significant.

## Results

### *In vitro* Activity of Omad Against Clinical *S. aureus* Isolates

Of the 127 MRSA isolates examined, 46 (36.22%), 80 (62.99%), and 1 (0.78%) were found to have Omad MIC levels of ≤0.25 mg/L (sensitive), 0.5 mg/L (sensitive), and 1 mg/L (intermediate), respectively. Of the 136 MSSA isolates examined, 23 (16.91%), 110 (80.88%), and 3 (2.20%) were categorized into these levels, respectively. Thus, there were higher frequencies of MSSA isolates than MRSA isolates with Omad MICs in the 0.5 and ≥1 mg/L levels. We analyzed the distribution of the above three Omad MIC levels among strains with sensitive and intermediate status relative to other common antibiotics (amikacin, erythromycin, ciprofloxacin, rifampicin, Tet, tobramycin, nitrofurantoin, quinupristin, and amoxicillin/clavulanate, vancomycin, and linezolid). The frequencies of isolates at each Omad MIC level found to be resistant to these antibiotics are reported in Table 1, together with the resistance breakpoints used. All *S. aureus* isolates in this study were susceptible to vancomycin and linezolid, and all of the MSSA isolates were susceptible to amoxicillin/clavulanate. Interestingly, as reported in Table 1, Tet-resistant MRSA were more frequent than Tet-resistant MSSA, and Omad MICs ≥ 0.5 mg/L were more frequent among MSSA isolates than among MRSA isolates, suggesting a non-conformity in the antimicrobial susceptibility dynamics of Tet and Omad. The characteristics of *S. aureus* with Omad MICs of 1 mg/L are summarized in Supplementary Table S4. Briefly, no genetic mutations in 30S ribosome units were detected and efflux pump inhibition reversed Omad resistance, as evidenced by significant reductions in MICs to ≤0.03 mg/L with CCCP and to 0.25–1 mg/L with PAβN.

**TABLE 1:** *Staphylococcus aureus* antibiotic resistance and correspondence to Omad MIC level.

**Class**	**Drug**	**Resistance rate (%)**	**Total *N***	**MIC breakpoint (mg/L)**	***N***	**Omad MIC level (mg/L), *N***		
						**≤0.25**	**0.5**	**1**
MRSA	Total		127	–	127	46	80	1
	Amikacin	51.61	124	≤16	60	24	36	0
				32	3	0	3	0
				≥64	61	20	42	1
	Erythromycin	99.21	127	≤0.5	1	1	0	0
				1–4	1	1	0	0
				≥8	125	44	80	1
	Ciprofloxacin	52.84	123	≤1	58	24	34	0
				2	1	0	1	0
				≥4	64	19	44	1
	Rifampicin	15.87	126	≤1	106	39	66	1
				≥4	20	6	14	0
	Tet	69.29	127	≤4	39	20	19	0
				8	15	6	9	0
				≥16	73	20	52	1
	Tobramycin	52.84	123	≤4	58	23	35	0
				≥16	65	21	43	1
	Nitrofurantoin	3.17	126	≤32	122	43	78	1
				64	2	1	1	0
				≥128	2	1	1	0
	Quinupristin	2.50	120	≤1	117	42	74	1
				2	1	0	1	0
				≥4	2	1	1	0
MSSA	Total		136	–	136	23	110	3
	Amikacin	5.30	132	≤16	124	21	100	3
				32	5	0	5	0
				≥64	2	0	2	0
	Erythromycin	83.58	134	≤0.5	22	1	21	0
				1–4	3	0	3	0
				≥8	109	21	85	3
	Ciprofloxacin	10.76	130	≤1	116	21	93	2
				≥4	14	0	13	1
	Rifampicin	4.51	133	≤1	127	21	104	2
				≥4	6	1	4	1
	Tet	50	136	≤4	68	21	47	0
				8	9	0	8	1
				≥16	59	2	55	2
	Tobramycin	45.60	125	≤4	68	14	52	2
				8	1	0	1	0
				≥16	56	5	51	0
	Nitrofurantoin	0.74	134	≤32	133	22	108	3
				64	1	0	1	0
	Quinupristin	2.5	120	≤1	116	20	94	2
				2	1	0	1	0
				≥4	2	0	2	0

### Omad MICs of *S. aureus* Isolates Harboring Tet-Resistance Genes

The frequencies of genes encoding the Tet-resistance factors Tet(M), Tet(L), Tet(K), and Tet(O), alone and in combination, in MRSA and MSSA isolates are reported in Supplementary Table S5. There were 109 *S. aureus* isolates harboring at least one Tet-resistance factor gene; their MIC_90_ values were consistently 0.5 mg/L. Omad exhibited excellent *in vitro* activity against both Tet-resistance gene carrying and non-carrying *S. aureus* isolates. Omad MICs for both MRSA and MSSA harboring Tet-resistance factors were ≤0.5 mg/L for all isolates, with the exception of three Tet(K) gene-carrying isolates (1 MRSA and 2 MSSA), indicating that overexpression of Tet(K) might impact Omad susceptibility. It is noteworthy that the Omad MIC values obtained for all 46 MSSA isolates carrying the Tet(K) gene, Tet(L) gene, or both were ≥0.5 mg/L. Meanwhile, of the 63 MRSA isolates with the Tet(M) gene, Tet(K) gene, Tet(L) gene, or some combination of these genes, just 47 (74.60%) had Omad MIC values ≥0.5 mg/L.

### Clonality of Omad MIC Distribution in Clinical *S. aureus* Isolates

MLST results for the 263 isolates are summarized in Supplementary Figure S3. To evaluate the relationship of ST clonality with Omad MIC distribution, we examined ST distributions relative to Tet and Omad MICs (Supplementary Table S6). Omad MICs ≥ 0.5 mg/L were found for 72.58% (45/62) of ST239-MRSA, 57.5% (23/40) of ST59-MRSA, and 57.14% (4/7) of ST1-MRSA isolates. Meanwhile, Omad MICs ≥ 0.5 mg/L were found for 89.66% (26/29) of ST7-MSSA, 89.47% (17/19) ST59-MSSA, 76.92% (10/13) of ST398-MSSA, 85.71% (6/7) of ST88-MSSA, and 71.43% (5/7) of ST120-MSSA isolates. Hence, Omad sensitivity differed between MRSA and MSSA of the same ST (e.g., ST59-MRSA vs. ST59-MSSA). MLST indicated that 72/81 (88.89%) MRSA isolates with Omad MICs ≥ 0.5 mg/L belonged to the top three MRSA STs (ST239, ST59, and ST1), whereas only 52/113 (46.12%) of MSSA isolates with Omad MICs ≥ 0.5 mg/L belonged to the top three MSSA STs (Supplementary Table S6 and Table 1), revealing a more pronounced clustering of Omad MIC creep in the top three MRSA STs than in the top three MSSA STs (nearly 90% vs. less than half).

### Omad Heteroresistance Frequency and Mechanism in *S. aureus*

Omad heteroresistance was identified in 0.0% (0/46) of MRSA with an Omad MIC ≤ 0.25 mg/L and 3.75% (3/80) of MRSA with an Omad MIC of 0.5 mg/L. Omad heteroresistance was identified in 0.0% (0/30) of MSSA with an Omad MIC ≤ 0.25 mg/L and 17.48% (18/103) of MSSA with an Omad MIC of 0.5 mg/L. We determined the Omad and Tig MICs of two clones from each heteroresistant subpopulation and found that their Omad MICs were in the range of 1–8 mg/L and their Tig MICs were in the range of 2–8 mg/L (shown in Supplementary Table S7 and data for six isolates subjected to *in vitro* resistance induction are shown in Table 2). Moreover, no genetic mutations were found in 30S ribosomal subunit genes (five 16SrRNA gene copies, 30S ribosomal protein S3 and S10 genes) in heteroresistance-derived clones (Table 2 and Supplementary Table S7). In efflux pump inhibition experiments, Omad MICs in heteroresistance-derived *S. aureus* clones were reduced to ≤0.03 mg/L by CCCP and reduced to 0.25–1 mg/L by PAβN (Supplementary Table S7).

**TABLE 2:** Antimicrobial susceptibility and resistance mechanism of seven groups of parental, heteroresistant, and Omad-induced resistant strains.

**Strain**	**MIC (mg/L)**		**Mutation(s)**								
	**Tig**	**Omad**	**RR1**	**RR2**	**RR3**	**RR4**	**RR5**	**S3**	**S10**	**RecB**	**FnBP**
CHS221 (S221)	0.5	0.5	W	W	W	W	W	W	W	W	W
CHS221-O (S221-O^∗^	4	8	W	W	W	W	W	W	W	W	W
CHS221-1Δ	32	32	W	A1124G	C810T	G1036A	G1248C	W	MeT48Ile	W	W
CHS221-2	32	32	G848T	A1124G	C810T	G1036A	A854C	W	MeT48Ile	W	W
					A1281G						
CHS165	0.5	0.5		W	W	W	W	W	W	W	W
CHS165-O^∗^	4	8	W	W	W	W	W	W	W	W	T672S,
CHS165-1Δ	32	32	T170G	G848A	C810T	G1036A	G1248C	W	LeT47His	W	T672S, I665V
				A1124G							
CHS165-2	32	32	T170G	G77A	C810T	G1036A	G742A	W	LeT47His	W	T672S, I665V
				A1124G	G848C		C1247T				
149	0.25	0.5	W	W	W	W	W	W	W	W	W
149-O^∗^	8	4	W	W	W	W	W	W	W	W	W
149-1 Δ	64	64	W	A1124G	C810T	G1036A	W	W	LeT47His	W	W
149-2	>64	128	W	A1124G	C810T	G1036A	C1247T	W	LeT47His	W	W
					G848C						
CHS759	0.25	0.5	W	W	W	W	W	W	W	W	W
CHS759-O^∗^	4	4	W	W	W	W	W	W	W	W	W
CHS759-1Δ	32	32	G1036A	A1124G	C810T	G185A	G1248C	W	Met48THr	W	I665V
				G1036A	G848C	G1036A	G1036A				
CHS759-2	32	32	W	A1124G	C810T	G185A	C1036T	W	Let47Let Tyr87His	W	I665V
				G1036A	A1281G	G1036A					
CHS810	0.25	0.5	W	W	W	W	W	W	W	W	W
CHS810-O^∗^	4	4	W	W	W	W	W	W	W	W	W
CHS810-1Δ	32	32	T170G	A1124G	C810T	G185A	T1257C	W	Tyr87His	W	W
						G1036A	G1248C				
CHS810-2	32	32	T170G	A1124G	C810T	G185A	A79G	W	Tyr87His	W	T672S,
						G1036A	T1257C				
CHS820	0.25	0.5	W	W	W	W	W	W	W	W	W
CHS820-O^∗^	4	2	W	W	W	W	W	W	W	W	W
CHS820-1Δ	64	128	T170G	A1124G	C810T	G185A	A79G	W	Tyr87His	W	W
						G1036A					
CHS820-2	64	128	T170G	A1124G	C810T	G185A	A79G	W	Tyr87His	W	W
			G848C		A1281G	G1036A	G848C				
MS4	0.125	0.125	W	W	W	W	W	W	W	W	W
MS4-O2	4	4	T170G	W	C810T	W	T1257C	W	W	W	T672S,
			G783A		A1281G						
MS4-O8	4	4	T170G	G848A	C810T	W	W	W	Asp60Tyr	RecB-M	T672S, I665V
			C1041T	T1124C	T1281C						

**FIGURE 1:**
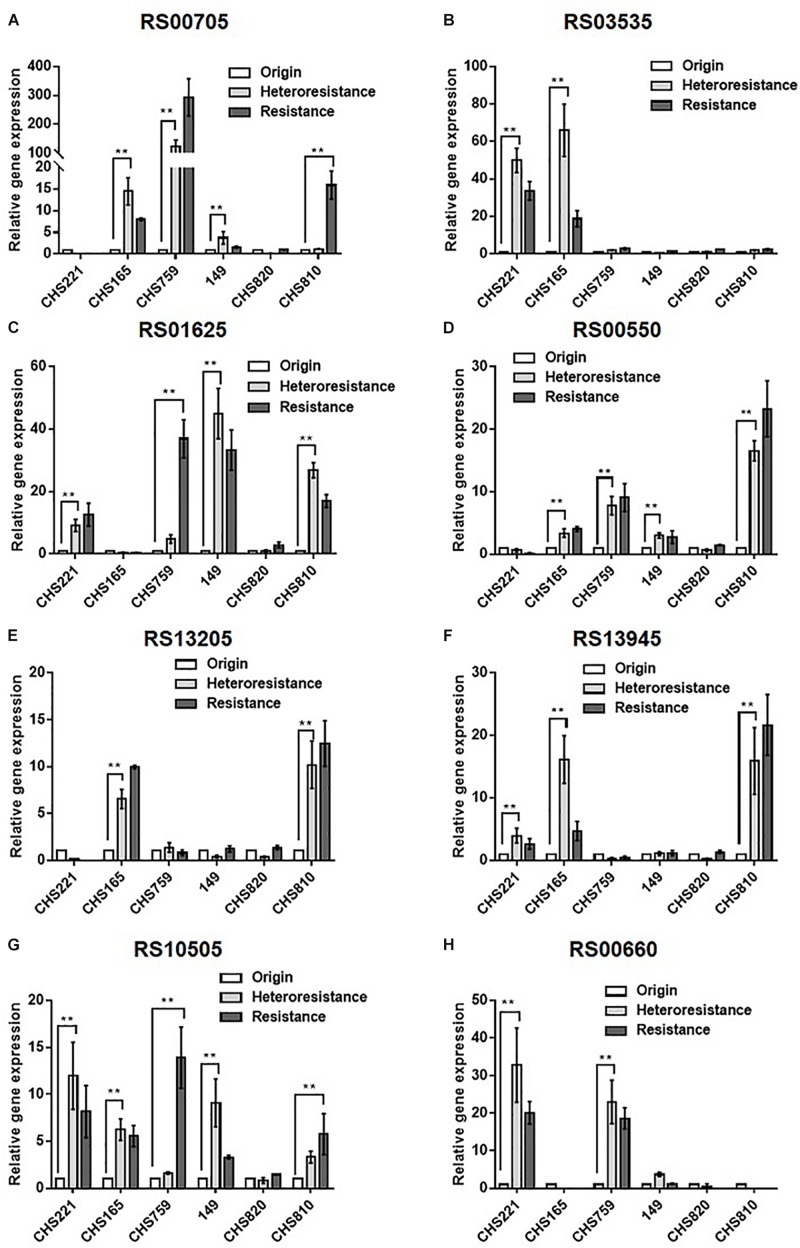
Comparison of the relative transcription of eight candidate DEGs among parental, heteroresistant derivative, and resistant isolates. Relative expression of USA300HOU_RS00705 **(A)**, USA300HOU_RS03535 **(B)**, USA300HOU_RS01625 **(C)**, USA300HOU_RS00550 **(D)**, USA300HOU_RS13205 **(E)**, USA300HOU_RS13945 **(F)**, USA300HOU_RS10505 **(G)**, and USA300HOU_RS00660 **(H)** were demonstrated by qRT-PCR analysis. The housekeeping gene *gyrB* was used as the endogenous reference gene. The original strain was used as the reference strain (expression = 1.0). All qRT-PCRs were carried out in triplicate. ^∗∗^*p* < 0.01, ^∗^*p* < 0.0.5. Parental strains are identified below the *X* axis and the relative folds increased are shown on the *Y* axis. The parental, heteroresistant, and resistant isolates are described in Table 2.

*Parental isolates are isolated with shading; ^∗^ and Δ represent heteroresistant and Omad-induced resistant strain, respectively (see qRT-PCR in Figure 1); RR1-7 are 16s rRNA gene copies; S3 and S10 are 30S ribosome proteins; W, wild-type (no mutation).*

### Association of Selected Candidate Genes With Omad Heteroresistance

Efflux pump inhibition experiments indicated that efflux pumps or membrane proteins might participate in the development of heteroresistance. Therefore, RNA-Seq was performed and unigene transcription levels were compared between the parental strain CHS221 (S221) and its heteroresistant derivative CHS221-O1 (S221-O). Ninety six DEGs were found by this approach between S221 and S221-O, including 58 upregulated and 38 down-regulated genes in the derivative strain (Supplementary Figure S4). KEGG pathway analysis showed that the pathways most frequently linked to DEGs were related to phosphate ion transport (3 DEGs), inorganic anion transport (3 DEGs), dihydrofolate reductase activity (2 DEGs), and glycine biosynthesis process (2 DEGs).

Subsequent qRT-PCRs for 30 candidate genes were carried out to test the accuracy of our transcriptomic analyses implicated eight efflux-pump encoding DEGs in heteroresistance. The expression levels of these eight candidate genes determined by RNA-Seq and qRT-PCR are shown in Table 3. The results of qRT-PCRs performed to quantitate transcription in six *S. aureus* strain groups—inclusive of parental strains, their heteroresistance derivatives, and resistant isolates (Table 2)—enabled us to probe the relationship of their expression levels with Omad susceptibility (Figure 1). The data suggest that transcription levels of the three candidate genes USA300HOU_RS03535, USA300HOU_ RS01625 (encodes a Na/Pi cotransporter family protein), and USA300HOU_RS00550 (encodes a branched-chain amino acid transport system II carrier protein) may impact heteroresistance occurrence.

**TABLE 3:** Transcriptional expression levels of eight DEGs between S221-O and S221 analyzed by RNA-Seq and qRT-PCR.

Gene_ID	Gene description	Relative increase in transcription in S221-O compared to S221	
		qRT-PCR	Fold change in RNA-Seq
USA300HOU _RS00705	Cell wall-anchored protein SasD	1.42 ± 0.12	2.73
USA300HOU _RS03535	Membrane protein	1.73 ± 0.14	2.83
USA300HOU _RS01625	Branched-chain amino acid transport system II carrier protein	2.77 ± 0.21	2.84
USA300HOU _RS00550	Na/Pi cotransporter family protein	1.18 ± 0.06	2.90
USA300HOU _RS13205	Amino acid permease	1.32 ± 0.11	2.97
USA300HOU _RS13945	PTS transporter subunit IIC	1.42 ± 0.07	3.29
USA300HOU _RS10505	hypothetical protein	5.73 ± 0.65	3.42
USA300HOU _RS00660	MFS transporter	3.27 ± 0.22	3.43

### Mechanism of Omad-Induced Resistance in *S. aureus* Under Omad Pressure

To evaluate Omad resistance mechanisms and Omad-Tig cross-resistance, *in vitro* induction experiments were carried out under Omad pressure with the following Omad-resistant *S. aureus*: CHS221 (MSSA), CHS165 (MSSA), 149 (MSSA), CHS759 (MRSA), CHS810 (MRSA), CHS820 (MRSA), and MS4. The Omad-resistant isolates were characterized with respect to MICs and resistance mechanisms (Table 2). Importantly, increasing Omad MICs were accompanied by increasing Tig MICs in Omad-resistant *S. aureus* isolates, indicating that Omad-Tig cross-resistance can be induced under Omad pressure. Moreover, upregulation of Omad MICs was related to increasing numbers of 16SrRNA copies with a genetic mutation. The mutation sites varied among the five 16SrRNA copies, with high frequencies of the T170G polymorphism in RR1, A1124G in RR2, C810T in RR3, and G1036A in RR4. Leu47His and Tyr87His amino acid substitutions in the 30S ribosomal protein S10 were relatively frequent in Omad-resistant bacteria.

### Candidate Genes Related to Omad Resistance in NGS

To identify the genetic mutations that correlate with Omad resistance, whole genome sequencing of MS4O8 was performed and variants relative to MS4 were detected by NGS in MUMmer, version 3.23 (Supplementary Table S8 and Table 4). Non-synonymous mutations were found in NI36_11090 (encodes 30S ribosomal protein S10), NI36_12460 (*fnbp* encoding FnBP protein), and NI36_ 00170 (*recB* encoding recombinase). Mutations affecting these three genes also emerged in our induced Omad-resistance experiment above (Table 2). Notably, 30S ribosomal protein S10 has been widely reported to be associated with Tet-class resistance and the impact of FnBP and RecB protein on Omad susceptibility need to be further verified.

**TABLE 4:** Non-synonymous mutations of candidate proteins correlated with Omad resistance found between MS4 and MS4O8.

**Gene_ID**	**Gene description**	**Amino acid mutations (non-syn) between MS4 and MS4O8**
NI36_11090	30S ribosomal protein S10	D60Y
NI36_12460	FnBP	T672S, I665V
NI36_00170	RecB	R10R, I23V, I23N, H24N, H24Q, V29L, V35M

### Relationship Between Candidate Genes Overexpression and Omad Susceptibility

The impacts of following candidate genes on Omad susceptibility in Omad-sensitive *S. aureus* isolates was conducted: USA300HOU_RS03535, USA300HOU_RS01625 (encodes a branched-chain amino acid transport system II carrier protein), USA300HOU_RS00550 (encodes a Na/Pi cotransporter family protein), *tet*(K), NI36_12460/NI36_12465 (*fnbp*), and NI36_00170 (*recB*). The former three were candidately implicated in Omad heteroresistance in our qRT-PCR experiments. Meanwhile, *tet*(K) was found frequently among *S. aureus* isolates with an Omad MIC of 1 mg/L, and the proteins encoded by *fnbp* and *recB* have been hypothesized to participate in antimicrobial resistance evolution.

The overexpression plasmids pRS00550, pRS01625, pRS03535, pTet(K), pRecB, pRecB-M, pFnBP, and pFnBP-M, where -M suffix indicates a mutated variant, were transformed into clinical isolates with low expression of the target gene (Supplementary Tables S9, S10). Stable overexpression of the candidate genes was confirmed by qRT-PCR (Supplementary Figure S5). The influence of the overexpression of these six genes on Omad susceptibility was reported in Table 5. Briefly, although RS00550, RS01625, and RS03535 did not elevate Omad or Tig MICs in the absence of antibiotic pressure, PAP experiments showed that RS00550 or RS01625 overexpression could lead to Omad heteroresistance compared with negative findings in controls (Table 5). RS00550 and RS01625 homology analysis results are reported in Supplementary Tables S11, S12.

**TABLE 5:** Omad and Tig MICs in *S. aureus* derivatives with overexpression of candidate genes and their impact on PAPs.

**Vector**	**Strain**	**MIC (mg/L)**				**PAP test positivity**
		**Parental isolates**		**Derivative isolates^∗^**		
		**Omad**	**Tig**	**Omad**	**Tig**	
pRS00550	SE7	0.25	0.5	0.25	0.5	10
	CHS545	0.5	0.5	0.25	0.5	12
	CHS569	0.25	0.5	0.25	0.5	5
pRS01625	SE4	0.25	0.5	0.25	0.5	3
	SE7	0.25	0.5	0.25	0.5	5
	CHS569	0.25	0.5	0.25	0.5	9
pRS03535	SE4	0.25	0.5	0.25	0.5	0
	SE7	0.25	0.5	0.25	0.5	0
	SE13	0.25	0.5	0.25	0.5	0
	CHS545	0.5	0.25	0.5	0.25	0
	CHS569	0.25	0.25	0.25	0.25	0
ptet(K)	SE4	0.25	0.5	0.5	0.5	0
	SE7	0.25	0.5	0.25	0.5	0
	SE13	0.25	0.5	0.25	0.5	0
	CHS545	0.5	0.25	0.25	0.5	0
	CHS569	0.25	0.25	0.25	0.5	0
pRecB	SE4	0.25	–	0.25	–	–
pRecB-M	SE4	0.25	–	0.25	–	–
pFBP	SE4	0.25	0.5	0.5	1	–
pFBP-M	SE4	0.25	0.5	1	1	–

*FBP-M has T672S and/or I665V mutations. RecB-M has R10R, I23V, I23N, H24N, H24Q, V29L, and/or V35M mutation. Positive PAP results are highlighted with shading.*

Overexpression of *tet*(*K*) did not elevate Omad or Tig MICs and had no apparent contribution to heteroresistance development in *S. aureus*. The overexpression of *fnbp* (NI36_12 460) and its mutated type led to MIC creep (Table 5), supporting the possibility that FnBPs may participate in Omad resistance development. Homology analysis results for *fnbp* are shown in Supplementary Table S13. Overexpression of *recB* and its mutants did not impact Omad susceptibility in *S. aureus*.

## Discussion

The presently observed low Omad MICs in this study support the supposition that Omad should be considered a prospective preferential choice for *S. aureus* infection treatment. Omad MICs ≥ 0.5 mg/L were more frequent among MSSA than MRSA in this study, and moreover, Tet-specific resistance genes, particularly *tet*(K) and *tet*(L), were found to be more common among MSSA than MRSA isolates, indicating that Omad may have greater efficacy against MRSA than MSSA. Our Omad MICs were higher than previously reported, perhaps due to regional variation and environmental factors (Villano et al., 2016; Pfaller et al., 2017a). We also obtained higher MICs for the new-generation Tet-class drug eravacycline in isolates from China than had been reported for isolates from the United States and Europe, suggesting that Tet-class drug MIC dynamics should monitored across global regions with particular attention to MIC creep in China (Zhang et al., 2018; Zheng et al., 2018).

Major mechanisms of Tet resistance in both Gram-positive and -negative microorganisms have been linked to ribosomal protection proteins and efflux pumps, most of which can be overcome with new generation tetracyclines, including Tig and Omad (Jones et al., 2006; Noel et al., 2012; Draper et al., 2014; Macone et al., 2014; Honeyman et al., 2015; Grossman, 2016; Heidrich et al., 2016; Villano et al., 2016; Pfaller et al., 2017b; Argudin et al., 2018; Zhang et al., 2018). In this study, we obtained low Omad MICs for *S. aureus*, even among isolates harboring a ribosomal protection protein, namely Tet(M), or an efflux pump factor, namely Tet(K) and Tet(L), uncovering an apparent advantage of using Omad to overcome Tet-specific resistance mechanisms, particularly those mediated by Tet(M), Tet(K), and Tet(L). Notwithstanding, Tig MICs were shown recently to be increased by a high transcriptional level of tet(M) and tet(L) in *Enterococcus faecium* (Fiedler et al., 2016). Because we observed a higher frequency of Tet-specific genes in MSSA than in MRSA from China and three isolates with MICs ≥ 1 mg/L harbored *tet*(K) in this study, we hypothesize that *tet*(K) overexpression may elevate Omad MICs, as was found in *E. coli* (Linkevicius et al., 2016). Our data demonstrate that *tet*(K) overexpression does not impact *S. aureus* susceptibility to Omad *in vitro*, and thus indicate that Omad can overcome the Tet(K)-mediated resistance in *S. aureus*.

Prior epidemiological data have revealed ST239 and ST59 to be predominant MRSA STs internationally, with MSSA ST predominance being more variable across regions (Atshan et al., 2013; Leon-Sampedro et al., 2016; Recker et al., 2017). *S. aureus* clonality of drug-resistance and virulence factors has been reported (Atshan et al., 2013; Leon-Sampedro et al., 2016; Recker et al., 2017). The present examination of the relationship of ST clonality with Omad susceptibility revealed a far higher frequency of the top-three MRSA STs with MICs ≥ 0.5 mg/L than of the top-three such MSSA STs. Although a definite relationship of ST clustering with Omad MICs has not been established, it is noteworthy that ST59-MSSA were much more likely to have Omad MICs ≥ 0.5 mg/L than were ST59-MRSA.

Heteroresistance frequency is an important harbinger of last-resort antibiotic resistance risk (Zhang et al., 2018; Zheng et al., 2018). The present findings of heteroresistance in 16.98% of MSSA and 3.75% of MRSA with Omad MICs ≥ 0.5 mg/L suggest a need to be alert to selection resistance under Omad pressure for *S. aureus*, especially in strains from China. Moreover, we observed relatively high Tig MICs for Omad heteroresistance-derived clones (2–8 mg/L), indicating a potential risk of Omad-Tig cross-resistance. Mutations affecting 30S ribosomal subunits, which have been reported to participate in Tet or Tig resistance, were not found in our heteroresistance-derived clones or clinical isolates with Omad MICs ≥ 1 mg/L, indicating that 30S ribosomal subunit mutations cannot explain Omad MIC creep and heteroresistance occurrence (Nguyen et al., 2014; Grossman, 2016; Argudin et al., 2018).

The progression of reduced Tig susceptibility in *S. aureus* has been linked with *mepR*/*mepA* encoded efflux pumps (Lupien et al., 2015). However, the possible role of efflux pumps and cell envelopes in Omad heteroresistance in *S. aureus* is unclear. Multiple reports have shown that protonophore efflux pump inhibitors (e.g., CCCP and PaβN) can be used to evaluate interactions between antibiotics and cell envelope components in bacteria (Klionsky et al., 2016; Bai et al., 2018; Yao et al., 2018). Here, we found that the efflux pump inhibitor PAβN and the cell envelope component inhibitor *CCCP* reduced Omad MICs of heteroresistance-derived *S. aureus* clones to as low as ≤0.03 mg/L and 0.12–1 mg/L, respectively, pointing to involvement of efflux pumps or cell envelopes in the progression of Omad heteroresistance in *S. aureus* (Klionsky et al., 2016; Bai et al., 2018). Omad MICs of isolates with Omad MICs ≥ 1 mg/L could also be reduced by CCCP and PAβN, supporting the notion that efflux pumps or cell envelopes may play an important role in reducing susceptibility to Omad. Our findings showing that overexpression of RS00550 or RS01625 can lead to Omad heteroresistance occurrence, despite having no impact on Omad MICs in the absence of Omad pressure, indicate that expression of genes can facilitate the formation of Omad resistance under antibiotic exposure. Our phylogenic analysis showed that both RS00550 and RS01625 encode efflux pump family proteins, supporting our hypothesis that efflux pump or membrane proteins contribute to Omad heteroresistance.

Crystallographic studies of the *Thermus thermophilus* 30S ribosomal subunit revealed at least one high-occupancy Tet-binding site and five other minor binding sites in 16S rRNA (Draper et al., 2014; Honeyman et al., 2015; Heidrich et al., 2016). Crystallographic studies with Tet, Tig, and Omad showed that, although they produced slightly different patterns of RNA cleavage and dimethylsulfate modification, all three antibiotics associated with the same binding site, albeit in somewhat different orientations. In several bacterial species, Tig and Omad have been shown to exhibit higher binding affinities and greater antitranslational potencies than Tet or minocycline, and 16SrRNA mutations have been shown to affect Tet binding sites in the 30S ribosomal subunit, which may confer Tet/Tig resistance (Nguyen et al., 2014; Grossman, 2016; Zheng et al., 2018). Consistent with previous reports, we found that greater numbers of 16S rRNA copies with genetic mutations were associated with higher levels Omad/Tig resistance in *S. aureus* isolates with Omad-resistance induced under Omad pressure (Nguyen et al., 2014; Grossman, 2016). This finding implicates the participation of 16SrRNA mutations in the development of the Omad resistance. Additionally, our finding of frequent 30S ribosomal protein S10 mutations in Omad-derived resistant isolates indicates that such mutations may be an important factor in Omad resistance evolution. It will be important to examine the unknown mechanism(s) underlying MIC elevation during Omad resistance evolution in *S. aureus*. In this study, NGS was performed to identify candidate genes that may be involved in Omad resistance development and FnBP was identified as a novel membrane molecule that may contribute to Omad MIC elevation. Mechanistically, we hypothesized that the overexpression of FnBP could alter the penetration potential of cell membranes.

## Conclusion

Omad exhibited excellent *in vitro* activity against clinical *S. aureus* isolates from China and might represent a preferential choice for the treatment of *S. aureus* infections. However, we must be alert to the potential risk of Omad heteroresistance in *S. aureus*, especially in strains with MICs ≥ 0.5 mg/L. Compared with MRSA, MSSA had relatively low MICs with a more facile tendency for the occurrence of Omad heteroresistance. Omad heteroresistance in both MSSA and MRSA could be reversed by CCCP and PaβN, indicating involvement of efflux pumps in Omad heteroresistance development in *S. aureus*. Furthermore, both RS01625 and RS00550, which encode efflux pump family proteins (a branched-chain amino acid transport system II carrier protein and an Na/Pi cotransporter family protein, respectively), were found to contribute to Omad heteroresistance. FnBP emerged as a novel molecule to be associated with Omad resistance and Omad MIC elevation in *S. aureus*. The present data contribute to understanding potential resistance mechanisms that may impact clinical applications of Omad.

## Data Availability Statement

All datasets generated for this study are included in the article/Supplementary Material.

## Author Contributions

QD and ZY initiated and designed the project. BB, ZP, and ZL performed the molecular biological experiments with bacteria. GX, XS, JZ, and ZC performed the PCRs. FZ, PL, and GX performed the bacterial culturing and MIC testing. All authors participated in the data analysis. QD, ZY, and BB wrote the manuscript incorporating comments from all authors.

## Conflict of Interest

The authors declare that the research was conducted in the absence of any commercial or financial relationships that could be construed as a potential conflict of interest.

## Footnotes

1 http://www.mlst.com/server

2 http://biobwa.sourceforge.net/
