# Supplementary material for: In vitro Activity and Heteroresistance of Omadacycline Against Clinical Staphylococcus aureus Isolates From China Reveal the Impact of Omadacycline Susceptibility by Branched-Chain Amino Acid Transport System II Carrier Protein, Na/Pi Cotransporter Family Protein, and Fibronectin-Binding Protein
Source: Front Microbiol. 2019 Nov 8;10:2546. doi: 10.3389/fmicb.2019.02546 (PMC6856048; doi:10.3389/fmicb.2019.02546)
Supplement: Supplementary file 1 [file Table_1.doc]

**Table S1 Primers used for the detection Tet-resistance genes, Tet target sites, RecB and FBP in *S aureus*** by PCR.

| **Target gene** | **Primer** | **Primer sequence (5'-3')** | **Amplicon size (bp)** | **Reference** |
| --- | --- | --- | --- | --- |
| *tet*(M) | tet(M)-F | CAATACAATAGGAGCAAGC | 974 | Nishimoto Y *et al*. 2005 |
|  | tet(M)-R | CGAACAAGAGGAAAGCATAAG |  |
| *tet*(L) | tet(L)-F | GTAACCAGCCAACTAATGAC | 908 | Nishimoto Y *et al*. 2005 |
|  | tet(L)-R | TTGGATCGATAGTAGCC |  |
| *tet*(K) | tet(K)-F | TCGATAGGAACAGCAGTA | 139 | Nishimoto Y *et al*. 2005 |
|  | tet(K)-R | CAGCAGATCCTACTCCTT |  |
| *tet*(O) | tet(O)-F | AACTTAGGCATTCTGGCTCAC | 515 | Nishimoto Y *et al*. 2005 |
|  | tet(O)-R | TCCCACTGTTCCATATCGTCA |  |
| 16SrRNA-RR1 | RR1-F | ATATGTCACGTTATTCCGCATCTTC | 2086 | In this study |
|  | RR1-R | GCGGTGTTTTGAGAGATTATTTA |  |  |
| 16SrRNA-RR2 | RR2-F | ATATGTCACGTTATTCCGCATCTTC | 2075 | In this study |
|  | RR2-R | GCAGACGCACAGGACTTC |  |  |
| 16SrRNA-RR3 | RR3-F | ATATGTCACGTTATTCCGCATCTTC | 1936 | In this study |
|  | RR3-R | GTCGTCAAACGGCACTAATA |  |  |
| 16SrRNA-RR4 | RR4-F | ATATGTCACGTTATTCCGCATCTTC | 1756 | In this study |
|  | RR4-R | ATCACCCGCTCCATAGATAAT |  |  |
| 16SrRNA-RR5 | RR5-F | ATATGTCACGTTATTCCGCATCTTC | 2345 | In this study |
|  | RR5-R | AGGTGCGATGGCAAAACA |  |  |
| SA30S-S3 | S3-R | GCAGATTCGATTTGACGAGAT | 810 | In this study |
|  | S3-F | ACGGTAAAGAAGAAGCTAAAG |  |  |
| SA-S10 | S10-R | CTCGAAAATAGTTGAACTGACTAAG | 1920 | In this study |
|  | S10-F | TTCAGAAGATTTCTCAGTGATTACG |  |  |
| RecB | RecB-F | GTGGTAAAAGTGATAAAAATGAAACG | 585 | In this study |
|  | RecB-R | CACGACCGGTGATTGCCTC |  |  |
| FBP | FBP-F | GTTACTACTTTACCTTGTTCCACTG | 1377 | In this study |
|  | FBP-R | TGACTCGTCTGCTAGTGATTTAAAC |  |  |

References: [1].Nishimoto Y, Kobayashi N, Alam MM, *et al*. Analysis of the prevalence of tetracycline resistance genes in clinical isolates of *Enterococcus faecalis* and *Enterococcus faecium*in a Japanese hospital. Microb Drug Resist. 2005 ;11:146-153.

**Table S2 Primer list used for** qRT-PCR

| **Primers name** | **Primer sequence (5'to3')** | **Amplicon size (bp)** | **Reference** |
| --- | --- | --- | --- |
| gyrB-F | ACATTACAGCAGCGTATTAG | 111 | In this study |
| gyrB-R | CTCATAGTGATAGGAGTCTTCT |  |  |
| RS00460-F | GTTTAGGTTGGTGGGATTT | 176 | In this study |
| RS00460-R | TCATTTCGTGTTGCTCTTAA |  |  |
| RS00915-F | GAGTGAACACAGAATTACAG | 114 | In this study |
| RS00915-R | TACGCATCAGGTATAACG |  |  |
| RS01680-F | CAGAAGATGTAGCAGAAG | 90 | In this study |
| RS01680-R | CTCCTTGTTCAGATATACC |  |  |
| RS01935-F | GGCTTCTACAACATCGTA | 126 | In this study |
| RS01935-R | TTACTTGTCTTCGTCTTCA |  |  |
| RS01965-F | TTAGAAGGAACATACACAGT | 136 | In this study |
| RS01965-R | CCATATACAAGCCATAGGA |  |  |
| RS04900-F | TGATAGAGTGGCAGTTATGTA | 112 | In this study |
| RS04900-R | AGGCATTGACGATAATAATCC |  |  |
| RS04910-F | AACACAGAAGATACACCAGAT | 167 | In this study |
| RS04910-R | GCAGACCAACCACTAAGT |  |  |
| RS08735-F | TACTGAAGAAGATAGCGATT | 191 | In this study |
| RS08735-R | ATTGAAGACCTGCCTTAA |  |  |
| RS10490-F | TTGGAACGCTTGTTATTAGT | 149 | In this study |
| RS10490-R | GACTCTACGCAATTCTGTAA |  |  |
| RS10520-F | TCTAATAATGTGAAATTCCCAGTA | 170 | In this study |
| RS10520-R | TCAGCAAGTCCTATGTCAT |  |  |
| RS10525-F | CATCAAGTTCGTTACACCAT | 184 | In this study |
| RS10525-R | TCTATATCACTAATACCTCTGTCT |  |  |
| RS13100-F | CATCAAGTTCGTTACACCAT | 184 | In this study |
| RS13100-R | TCTATATCACTAATACCTCTGTCT |  |  |
| RS00705-F | GACGCAAGCACAATATAC | 181 | In this study |
| RS00705-R | GCCATTCATATCTCTAACAG |  |  |
| RS03535-F | TGCCTCAATTCCTACATT | 190 | In this study |
| RS03535-R | ATAACCTGCTACCATCATT |  |  |
| RS01625-F | AATATGTGGATTGGTATGC | 113 | In this study |
| RS01625-R | ATACGATTGCCTACACTT |  |  |
| RS13600-F | GGATTAGCATCACAGAAT | 142 | In this study |
| RS13600-R | CAGCCATTACATTAGGAA |  |  |
| RS00550-F | TCCAGTATTAGGTGTTATTG | 153 | In this study |
| RS00550-R | GTTGTTCCGATATTAGCA |  |  |
| RS13205-F | ATGTTGGATTGTATCAAGTAT | 139 | In this study |
| RS13205-R | TCTGGCACCTAATAAGTT |  |  |
| RS05060-F | TTGCTGTAACACTTCCTAA | 90 | In this study |
| RS05060-F | CACCAATCATTCCACCTA |  |  |
| RS12375-F | GCATCATCGCCTATACTT | 137 | In this study |
| RS12375-R | AACAATATCTAATCCTGACTTATC |  |  |
| RS13945-F | TAGTATGGCATTGATTGTATTCC | 107 | In this study |
| RS13945-R | TATTACCGATGGCTGTTGT |  |  |
| RS10505-F | TGGTTATTATTTACAAGCAACTAA | 108 | In this study |
| RS10505-R | CGTCGATCATTACTTCTACTTA |  |  |
| RS00660-F | GTATTGGCATCTGACTTA | 177 | In this study |
| RS00660-R | TTGAATAGTGGTAGTATCTG |  |  |
| RS00495-F | GGTGGCGTAAGATACTATT | 161 | In this study |
| RS00495-R | CTTTCCGTTATATTTGTATTGTC |  |  |
| RS12525-F | ATTGGAGAATTAGGTAAGAAGTT | 130 | In this study |
| RS12525-R | TGATGGATAGTATGAGATATGGA |  |  |
| RS09460-F | GCAGTGTTATTAACTCAAT | 130 | In this study |
| RS09460-R | ACAATTAGAATAATCCAAGG |  |  |
| RS10445-F | GACAAGTTATGAAGAAGAA | 146 | In this study |
| RS10445-R | CGATAATGTATTGTTGAATC |  |  |
| RS15445-F | TTATACGGGAATGCTAAAG | 114 | In this study |
| RS15445-R | TTACATCTATCCTTGTTCAC |  |  |
| RS14685-F | AAGTTATGTATGTCTGGTAAGC | 108 | In this study |
| RS14685-R | GGAACGAGATGGTTATTCAC |  |  |
| RS15095-F | AGTAGTTAAAGTTCTCCCAAG | 166 | In this study |
| RS15095-R | TTTATCTTCAGTATATTCCCGTAT |  |  |

**Table S3 primers list used for the vector constructs** in this study

| **Primers** | | **Sequences（5’-3’）** | **Product**  **Length (bp)** | | **Underline** |
| --- | --- | --- | --- | --- | --- |
| **Construction of theoverexpression plasmids** | | | | | |
| ORS00550-F | CGCGGATCCGATTTCAAATCATGAGACTGG | | | 2123 | BamHI |
| ORS00550-R | CCGGAATTCCTTACCGCATACTAATGAAGC | | |  | EcoRI |
| ORS00705-F | CGCGGATCCTGTGTAATGGGATAGCACGTA | | | 1108 | BamHI |
| ORS00705-R | CCGGAATTCTGCCAATATAACATTAGCGAG | | |  | EcoRI |
| ORS01625-F | CGCGGATCCTAGCAGTCCTCACTCATACAA | | | 1653 | BamHI |
| ORS01625-R | CCGGAATTCTAGACAGACGGTATCACTGAA | | |  | EcoRI |
| OtetK-F | CGCGGATCCTTTAGTGTATTAAATGAAATGGT | | | 1326 | BamHI |
| OtetK-R | CCGGAATTCCTATTCAAACTGCTTTTCAGAACG | | |  | EcoRI |
| **Verification of theoverexpression plasmids** | | | | | |
| IDRS00550-F | TTCGCATTACAGAAGATGCAC | | 828 | |  |
| IDRS00550-R | CGCCTGTCACTTTGCTTGATA | |  |  |  |
| IDRS00705-F | CGAATGTAGATGCAAATGGTG | | 749 | |  |
| IDRS00705-R | GCGCCTGTCACTTTGCTTGAT | |  |  |  |
| IDRS01625-F | CCGTGTCGATTGCACTTGTAC | | 803 | |  |
| IDRS01625-R | CTTGTTGCTGTTCCTGTTCTG | |  |  |  |
| IDtetK-F | TGCTTCTGGAATGAGTTTGCT | | 619 | |  |
| IDtetK-R | GTTTCCTGCTAAACCATTTAG | |  |  |  |

**Table S4. Characteristics of *S. aureus* with Omad MIC of 1 mg/L**

| No. | MIC (mg/L) and Mol;ecular characteristics of *S aureus* | | | | | | |  | Genetic mutations in 30S ribosomal subunits∆ | | | | | | |
| --- | --- | --- | --- | --- | --- | --- | --- | --- | --- | --- | --- | --- | --- | --- | --- |
|  | Species | Omad  MIC | ST | Tetra  ^※^ | Tet# | Omad  +CCP | Omad  +PAβN |  | RR1 | RR2 | RR3 | RR4 | RR5 | S3 | S10 |
| 1 | MSSA | 1 | ST7 | >8 | Tet(K) | ≤0.03 | 1 |  | - | - | - | - | - | - | - |
| 2 | MSSA | 1 | ST59 | >8 | Tet(K) | ≤0.03 | 0.5 |  | - | - | - | - | - | - | - |
| 3 | MSSA | 1 | ST239 | >8 | - | ≤0.03 | 0.5 |  | - | - | - | - | - | - | - |
| 4 | MRSA | 1 | ST239 | >8 | Tet(K) | ≤0.03 | 0.5 |  | - | - | - | - | - | - | - |

∆, 30S ribosomal subunits included the five copies of 16S ribosomal genes and 30S ribosomal protein S3 and S10. #, - represent the negative Tet-specific genes and negative mutation was found in 30S ribosome units respectively.

**Table S5**. In vitro activity of Omad against Tet-resistant strains.

| **Class** | **Tet resistance factor(s)** | **No. isolates** |  | **Tet MIC level (mg/L)** | | |  | **Omad MIC level**  **(mg/L)** | | |  | **Omad MIC range (mg/L)** | **Omad MIC_50_/_90_ (mg/L)** |
| --- | --- | --- | --- | --- | --- | --- | --- | --- | --- | --- | --- | --- | --- |
|  |  |  |  | **≤4** | **8** | **≥16** |  | **≤0.25** | **0.5** | **≥1** |  |  |  |
| MRSA | Tet(M) | 25 |  | 0 | 1 | 24 |  | 6 | 19 | 0 |  | 0. 25–0.5 | 0.5/0.5 |
|  | Tet(K) | 23 |  | 1 | 6 | 16 |  | 8 | 14 | 1 |  | 0.125–1 | 0.5/0.5 |
|  | Tet(L) | 5 |  | 0 | 1 | 4 |  | 1 | 4 | 0 |  | 0.25–0.5 | 0.5/0.5 |
|  | Tet(M), Tet(L) | 4 |  | 0 | 1 | 3 |  | 0 | 4 | 0 |  | 0.5 | 0.5/0.5 |
|  | Tet(M), Tet(K) | 2 |  | 0 | 0 | 2 |  | 0 | 2 | 0 |  | 0.5 | - |
|  | Tet(L), Tet(K) | 3 |  | 0 | 3 | 0 |  | 0 | 3 | 0 |  | 0.5 | 0.5/0.5 |
|  | Tet(M), Tet(L), Tet(K) | 1 |  | 0 | 0 | 1 |  | 1 | 0 | 0 |  | 0.125 | - |
|  | -^a^ | 64 |  | 38 | 3 | 23 |  | 30 | 34 | 0 |  | 0.05–0.5 | 0.5/0.5 |
| MSSA | Tet(K) | 39 |  | 2 | 8 | 29 |  | 0 | 37 | 2 |  | 0.5–1 | 0.5/0.5 |
|  | Tet(L) | 2 |  | 0 | 0 | 2 |  | 0 | 2 | 0 |  | 0.5 | - |
|  | Tet(L), Tet(K) | 5 |  | 1 | 0 | 4 |  | 0 | 5 | 0 |  | 0.5 | 0.5/0.5 |
|  | -^a^ | 90 |  | 65 | 1 | 24 |  | 23 | 66 | 1 |  | 0.0625–1 | 0.5/0.5 |

- ^a^ negative for Tet resistance genes; Tet(O) was not found in any of the isolates.

**Table S6. ST associations with Omad MIC distribution of *S. aureus* isolates.**

| **Class** | **ST*** | **Total**  **N** | **Tet MIC (mg/L), N** | | |  | **Omad MIC (mg/L), N** | | |
| --- | --- | --- | --- | --- | --- | --- | --- | --- | --- |
|  |  |  | **≤4** | **8** | **≥16** |  | **≤0.25** | **0.5** | **≥1** |
| MRSA | ST239 | 62 | 6 | 3 | 53 |  | 17 | 44 | 1 |
|  | ST59 | 40 | 18 | 12 | 10 |  | 17 | 23 | 0 |
|  | ST1 | 7 | 6 | 0 | 1 |  | 3 | 4 | 0 |
| MSSA | ST7 | 29 | 8 | 1 | 20 |  | 3 | 25 | 1 |
|  | ST59 | 19 | 9 | 5 | 5 |  | 2 | 16 | 1 |
|  | ST398 | 13 | 5 | 0 | 8 |  | 3 | 10 | 0 |
|  | ST88 | 7 | 3 | 0 | 4 |  | 1 | 6 | 0 |
|  | ST120 | 7 | 5 | 0 | 2 |  | 2 | 5 | 0 |

***,showed that with ST number of N≥7.**

**Table S7 Characteristics of heteroresistance derivative *S.aureus* isolates.**

| NO. | Characteristics of the parental isolates | | | |  | Characteristics and mechanism of Heteroresistance-derived clones | | | | | | |
| --- | --- | --- | --- | --- | --- | --- | --- | --- | --- | --- | --- | --- |
|  | Omad  MIC(mg/L) | ST | Tetra  MIC(mg/L)※ | tet# |  | NO＊ | MIC(mg/L) | | | |  | Genetic mutation  in 30S ribosomal  subunits^∆^ |
|  |  |  |  |  |  |  | Omd | Tig | Omad  +CCP | Omad  +PAβN |  |  |
| 1 | 0.5 | ST7 | - | tetk |  | CHS221-O | 8 | 4 | ≤0.03 | 1 |  | - |
|  |  |  |  |  |  | CHS221-H2 | 8 | 4 | ≤0.03 | 0.5 |  | - |
| 2 | 0.5 | ST398 | >8 | tetk |  | CHS165-O | 8 | 4 | ≤0.03 | 0.5 |  | - |
|  |  |  |  |  |  | CHS165-H2 | 2 | 4 | ≤0.03 | 0.5 |  | - |
| 3* | 0.5 | ST239 | >8 | no |  | CHS759-O | 4 | 4 | ≤0.03 | 0.5 |  | - |
|  |  |  |  |  |  | CHS759-H2 | 2 | 4 | ≤0.03 | 0.5 |  | - |
| 4* | 0.5 | ST239 | >8 | tetm |  | CHS820-O | 2 | 4 | ≤0.03 | 0.5 |  | - |
|  |  |  |  |  |  | CHS820-H2 | 2 | 4 | ≤0.03 | 0.5 |  | - |
| 5* | 0.5 | ST59 | 8 | tetl |  | CHS810-O | 4 | 4 | ≤0.03 | 1 |  | - |
|  |  |  |  |  |  | CHS810-H2 | 4 | 4 | ≤0.03 | 0.5 |  | - |
| 6 | 0.5 | ST7 | ≤0.5 | - |  | 149-O | 4 | 8 | ≤0.03 | 0.5 |  | - |
|  |  |  |  |  |  | 149-H2 | 4 | 8 | ≤0.03 | 0.5 |  | - |
| 7 | 0.5 | ST59 | >8 | tetk |  | CHS606-H1 | 4 | 4 | ≤0.03 | 1 |  | - |
|  |  |  |  |  |  | CHS606-H2 | 4 | 4 | ≤0.03 | 1 |  | - |
| 8 | 0.5 | ST59 | >8 | - |  | CHS619-H1 | 2 | 4 | ≤0.03 | 1 |  | - |
|  |  |  |  |  |  | CHS619-H2 | 2 | 4 | ≤0.03 | 1 |  | - |
| 9 | 0.5 | ST398 | >8 | - |  | CHS62-H1 | 2 | 2 | ≤0.03 | 1 |  | - |
|  |  |  |  |  |  | CHS62-H2 | 2 | 4 | ≤0.03 | 1 |  | - |
| 10 | 0.5 | NT | ≤0.5 | - |  | SE14-H1 | 2 | 2 | ≤0.03 | 1 |  | - |
|  |  |  |  |  |  | SE14-H2 | 2 | 2 | ≤0.03 | 0.5 |  | - |
| 11 | 0.5 | ST1 | 8 | tetk |  | CHS46-H1 | 4 | 8 | ≤0.03 | 0.5 |  | - |
|  |  |  |  |  |  | CHS46-H2 | 4 | 8 | ≤0.03 | 0.5 |  | - |
| 12 | 0.5 | ST7 | >8 | - |  | CHS150-H1 | 2 | 4 | ≤0.03 | 0.5 |  | - |
|  |  |  |  |  |  | CHS150-H2 | 2 | 2 | ≤0.03 | 0.5 |  | - |
| 13 | 0.5 | ST6 | >8 | tetk |  | CHS49-H1 | 2 | 4 | ≤0.03 | 1 |  | - |
|  |  |  |  |  |  | CHS49-H2 | 2 | 4 | ≤0.03 | 1 |  | - |
| 14 | 0.5 | ST59 | >8 | tetk |  | CHS447-H1 | 4 | 8 | ≤0.03 | 1 |  | - |
|  |  |  |  |  |  | CHS447-H2 | 2 | 4 | ≤0.03 | 1 |  | - |
| 15 | 0.5 | ST120 | >8 | tetk |  | CHS144-H1 | 4 | 4 | ≤0.03 | 0.5 |  | - |
|  |  |  |  |  |  | CHS144-H2 | 4 | 2 | ≤0.03 | 0.5 |  | - |
| 16 | 0.5 | ST120 | >8 | - |  | CHS582-H1 | 2 | 4 | ≤0.03 | 0.25 |  | - |
|  |  |  |  |  |  | CHS582-H2 | 2 | 4 | ≤0.03 | 0.25 |  | - |
| 17 | 0.5 | ST633 | >8 | tetk |  | CHS95-H1 | 4 | 4 | ≤0.03 | 0.5 |  | - |
|  |  |  |  |  |  | CHS95-H2 | 4 | 4 | ≤0.03 | 0.5 |  | - |
| 18 | 0.5 | ST7 | 8 | tetk |  | CHS364-H1 | 4 | 4 | ≤0.03 | 0.12 |  | - |
|  |  |  |  |  |  | CHS364-H2 | 4 | 4 | ≤0.03 | 0.12 |  | - |
| 19 | 0.5 | ST5 | >8 | tetk |  | CHS91-H1 | 4 | 4 | ≤0.03 | 0.5 |  | - |
|  |  |  |  |  |  | CHS91-H2 | 2 | 4 | ≤0.03 | 0.5 |  | - |
| 20 | 0.5 | ST239 | >8 | tem |  | CHS666-H1 | 4 | 4 | ≤0.03 | 1 |  | - |
|  |  |  |  |  |  | CHS666-H2 | 4 | 4 | ≤0.03 | 1 |  | - |
| 21 | 0.5 | ST7 | >8 | tetk |  | CHS259-H1 | 2 | 4 | ≤0.03 | 0.5 |  | - |
|  |  |  |  |  |  | CHS259-H2 | 2 | 4 | ≤0.03 | 0.5 |  | - |

＊, represented the number of heteroresistance-derive clines; ∆, represented 30S ribosomal subunits included the five copies of 16S ribosomal genes and 30S ribosomal protein S3 and S10. ※ represent the Tet MIC of the parental *S aureus*. * MRSA, all others are MSSA. #, represented the detection of Tet specific resistance genes. - , represent the negativeresults by PCR. Shadow represented that these parental isolates were further selected under Omad pressure in vitro.

**Table S8 List of all nucleotide and amino acid mutations by comparison of whole genome between MS4 and MS4O8**

| Gene NO | Reference_  CP009828 | MS4O8 | ref_base<->  sample_base | proPos | ref_codon<->  sample_codon | ref_aa<->  sample_aa | mutate_  type | ref_gene_ID | ref_gene_  start | ref_gene  _end |
| --- | --- | --- | --- | --- | --- | --- | --- | --- | --- | --- |
| CP009828_2184774 | C | A | C<->A | 60 | GAT<->TAT | D<->Y | nonsyn | NI36_11090 | 2184643 | 2184951 |
| CP009828_2454732 | G | C | G<->C | 672 | ACC<->AGC | T<->S | nonsyn | NI36_12460 | 2453882 | 2456746 |
| CP009828_2454754 | T | C | T<->C | 665 | ATT<->GTT | I<->V | nonsyn | NI36_12460 | 2453882 | 2456746 |
| CP009828_2458142 | G | A | G<->A | 819 | ATC<->ATT | I<->I | syn | NI36_12465 | 2457428 | 2460598 |
| CP009828_41271 | A | G | A<->G | - | - | - | - | intergenic | -- | -- |
| CP009828_41338 | C | T | C<->T | 10 | CGC<->CGT | R<->R | syn | NI36_00170 | 41309 | 42985 |
| CP009828_41350 | A | T | A<->T | 14 | TCA<->TCT | S<->S | syn | NI36_00170 | 41309 | 42985 |
| CP009828_41359 | G | A | G<->A | 17 | TCG<->TCA | S<->S | syn | NI36_00170 | 41309 | 42985 |
| CP009828_41362 | G | A | G<->A | 18 | GAG<->GAA | E<->E | syn | NI36_00170 | 41309 | 42985 |
| CP009828_41368 | G | T | G<->T | 20 | GGG<->GGT | G<->G | syn | NI36_00170 | 41309 | 42985 |
| CP009828_41371 | C | T | C<->T | 21 | TAC<->TAT | Y<->Y | syn | NI36_00170 | 41309 | 42985 |
| CP009828_41374 | A | T | A<->T | 22 | TCA<->TCT | S<->S | syn | NI36_00170 | 41309 | 42985 |
| CP009828_41375 | A | G | A<->G | 23 | ATC<->GTC | I<->V | nonsyn | NI36_00170 | 41309 | 42985 |
| CP009828_41376 | T | A | T<->A | 23 | ATC<->AAC | I<->N | nonsyn | NI36_00170 | 41309 | 42985 |
| CP009828_41377 | C | A | C<->A | 23 | ATC<->ATA | I<->I | syn | NI36_00170 | 41309 | 42985 |
| CP009828_41378 | C | A | C<->A | 24 | CAT<->AAT | H<->N | nonsyn | NI36_00170 | 41309 | 42985 |
| CP009828_41380 | T | G | T<->G | 24 | CAT<->CAG | H<->Q | nonsyn | NI36_00170 | 41309 | 42985 |
| CP009828_41393 | G | C | G<->C | 29 | GTA<->CTA | V<->L | nonsyn | NI36_00170 | 41309 | 42985 |
| CP009828_41411 | G | A | G<->A | 35 | GTG<->ATG | V<->M | nonsyn | NI36_00170 | 41309 | 42985 |

**Table S9 The transcriptional expression level of four candidate genes (RS00550, RS01625, RS03535 and tet(K)) in fifteen clinical *S aureus* isolates for transformation.**

| **Strain NO** | **RS00550** | **RS01625** | **RS03535** | ***tet(K)*** |
| --- | --- | --- | --- | --- |
| CHS548 | 1.270467 | 0.000373 | 0 | 0.000268 |
| SE6 | 0.585515 | 0.252126 | 0.003005 | 0.024594 |
| SE8 | 2.318645 | 0.172583 | 0.003333 | 0.01929 |
| CHS569 | 0.509343 | 0.000192 | 0.004878 | 7.43E-05 |
| SE9 | 0.99827 | 0.291079 | 0.009084 | 0.537313 |
| CHS545 | 0.695838 | 0.276068 | 0.009487 | 0.271752 |
| CHS568 | 5.521373 | 0.752791 | 0.025102 | 3.246137 |
| SE4 | 0.796173 | 2.36E-05 | 0.030545 | 0.191715 |
| SE5 | 1.245925 | 0.30541 | 0.039366 | 0.4831 |
| SE2 | 1.060896 | 3.03E-05 | 0.051078 | 0.140537 |
| SE11 | 0.977707 | 0.211709 | 0.057444 | 0.188067 |
| SE14 | 0.338918 | 0.221855 | 0.059335 | 0.290996 |
| SE7 | 1.392961 | 0.484075 | 0.071795 | 0.29091 |
| SE13 | 0.852705 | 7.78E-05 | 0.075944 | 0.417457 |
| CHS563 | 1.615725 | 0.298696 | 0.202307 | 0.269879 |

**Table S10 Transformation of four candidate genes in *S aureus* clinical isolate**s.

| Gene-NO | Strain1 | Strain 2 | Strain 3 | Strain 4 | Strain 5 |
| --- | --- | --- | --- | --- | --- |
| RS00550 | CHS545 | CHS569 | - | SE7 | - |
| RS01625 | - | CHS569 | SE4 | SE7 | - |
| RS03535 | CHS545 | CHS569 | SE4 | SE7 | SE13 |
| tet(K) | CHS545 | CHS569 | SE4 | SE7 | SE13 |

-, represents no transformation of this strain.

**Table S11. Phylogeny and homology of USA300HOU_RS00550** protein.

| **Strain** | ***S. aureus* USA300_TCH1516ABX28153.1** | | | |
| --- | --- | --- | --- | --- |
|  | **GenBank ID** | **No. residues** | **Identity %** | **Description or predicted function** |
| *S. aureus USA300_FPR3757* | ABD20376.1 | 553 | 99.6 | Na/Pi cotransporter family protein |
| *S. aureus strain NCTC12233* | VDZ13514.1 | 555 | 100 | Na/Pi cotransporter II-related protein |
| *S. aureus str. Newman* | BAF66321.1 | 555 | 100 | conserved hypothetical protein |
| *S. aureus subsp. aureus N315* | BAB41319.1 | 555 | 100 | conserved hypothetical protein |
| *Streptococcus pneumoniae* | COD94233.1 | 540 | 66.4 | Na/Pi cotransporter II-like protein |
| *S. pneumoniae GA16833* | EHE06208.1 | 536 | 62.2 | Na+/Pi-cotransporter family protein |
| *Klebsiella pneumoniae* | PCQ20187.1 | 548 | 76.4 | Na/Pi cotransporter |
| *Escherichia coli* | WP_086629032.1 | 516 | 40.8 | Na/Pi cotransporter family protein |
| *Acinetobacter baumannii* | SST03618.1 | 527 | 40.9 | sodium-dependent inorganic phosphate (Pi) transporter |

**Table S12. Phylogeny and homology of USA300HOU_ RS01625** protein.

| **Strain** | ***S. aureus* USA300_TCH1516ABX28355.1** | | | |
| --- | --- | --- | --- | --- |
|  | **GenBank ID** | **No. residues** | **Identity %** | **Description or predicted function** |
| *S. aureus strain UTSW MRSA 55* | AMO15703.1 | 435 | 100 | branched-chain amino acid transporter II carrier protein |
| *S. aureus strain NCTC12233* | VDZ13710.1 | 435 | 100 | branched-chain amino acid transport system carrier protein |
| *S. aureus str. Newman* | BAF66520.1 | 435 | 100 | branched-chain amino acid transport system II carrier protein |
| *S. aureus strain 2395 USA500* | AIL56785.1 | 435 | 100 | branched-chain amino acid transporter II carrier protein |
| *Staphylococcus epidermidis* | WP_049392738.1 | 439 | 53.6 | branched-chain amino acid transport system II carrier protein |
| *E. faecalis* | WP_104877089.1 | 424 | 51.8 | branched-chain amino acid transport system II carrier protein |
| *S. pneumoniae* | CKF10693.1 | 425 | 59 | branched-chain amino acid transport system II carrier protein |
| *Streptococcus agalactiae* | CCO74972.1 | 421 | 51.6 | branched-chain amino acid transporter |
| *K. pneumoniae* | PCQ21257.1 | 422 | 51.7 | branched-chain amino acid transport system II carrier protein |
| *E. coli* | WP_021567273.1 | 421 | 54.2 | branched-chain amino acid transporter carrier protein BrnQ |
| *A. baumannii* | SST09302.1 | 423 | 54.5 | branched chain amino acid transporter |

**Table S13. Phylogeny and homology of NI36_12460 protein.**

| **Strain** | ***Staphylococcus aureus* strain MS4 ALR00607.1** | | | |
| --- | --- | --- | --- | --- |
|  | **GenBank ID** | **No. residues** | **Identity %** | **Description or predicted function** |
| *S. aureus subsp. aureus M013* | AEV79458.1 | 931 | 97.6 | FnBP |
| *S. aureus subsp. aureus 21331* | EHM74289.1 | 928 | 92.8 | FnBP-A |
| *S. aureus* | KIT91245.1 | 935 | 93.9 | fFnBP |
| *S. aureus* | WP_064138954.1 | 959 | 92.6 | FnBB |
| *S. epidermidis* | PVU43655.1 | 183 | 18 | FnBP, partial |
| *E. faecalis* | WP_126259823.1 | 319 | 11.4 | LPXTG cell wall anchor domain-containing protein |
| *S. pneumoniae* | WP_061367431.1 | 111 | 2.9 | YSIRK-type signal peptide-containing protein |
| *S. agalactiae* | WP_047205529.1 | 143 | 4 | YSIRK-type signal peptide-containing protein |
