# Supplementary figures and images for: In vitro Activity and Heteroresistance of Omadacycline Against Clinical Staphylococcus aureus Isolates From China Reveal the Impact of Omadacycline Susceptibility by Branched-Chain Amino Acid Transport System II Carrier Protein, Na/Pi Cotransporter Family Protein, and Fibronectin-Binding Protein
Source: Front Microbiol. 2019 Nov 8;10:2546. doi: 10.3389/fmicb.2019.02546 (PMC6856048; doi:10.3389/fmicb.2019.02546)

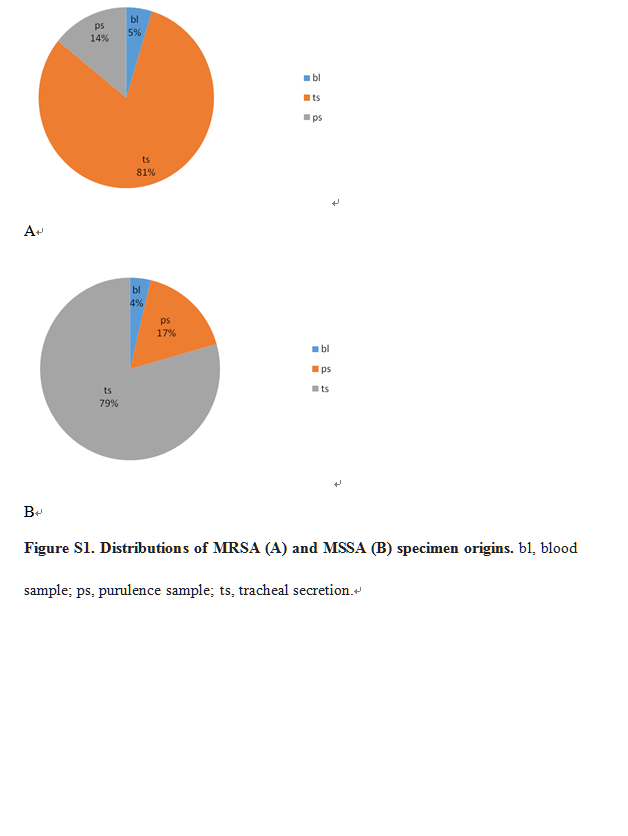

Supplement: Supplementary file 3 [file Image_1.PNG]

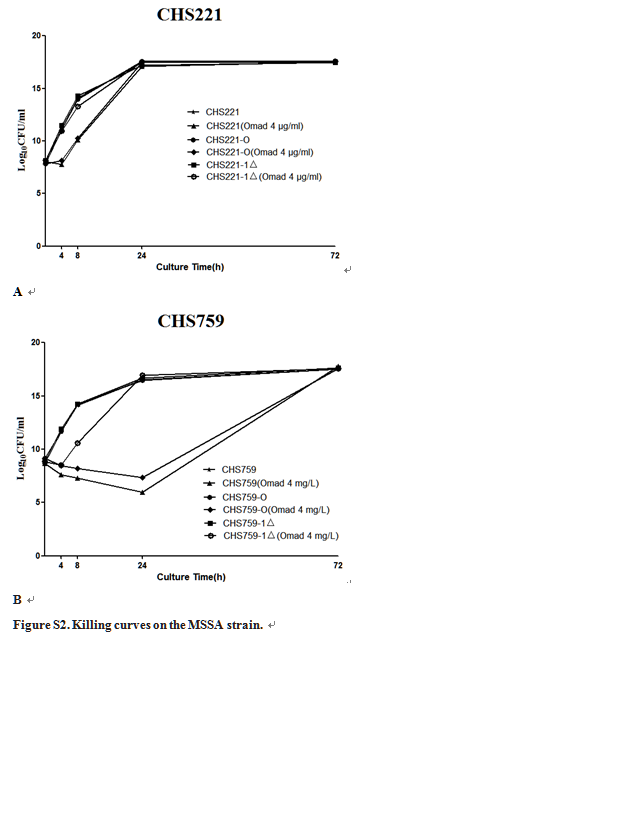

Supplement: Supplementary file 4 [file Image_2.PNG]

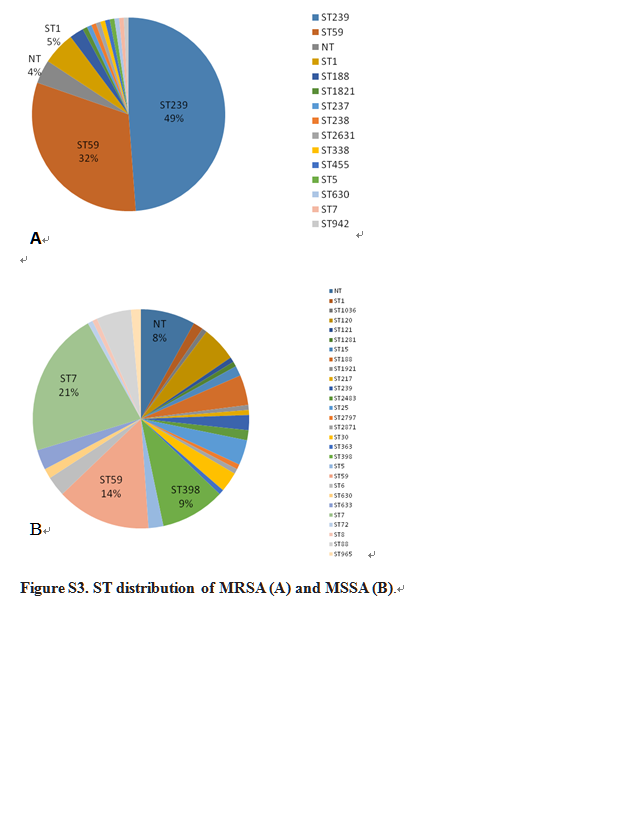

Supplement: Supplementary file 5 [file Image_3.PNG]

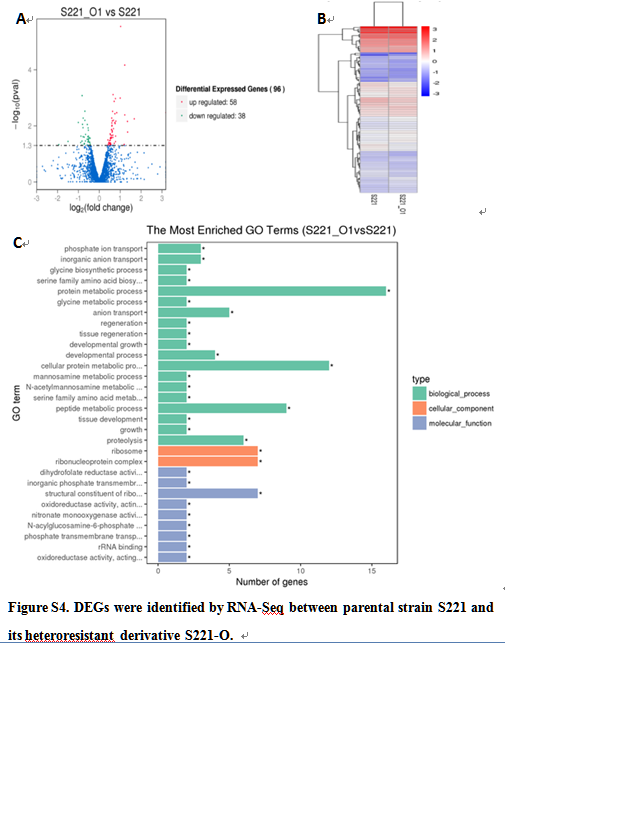

Supplement: Supplementary file 6 [file Image_4.PNG]

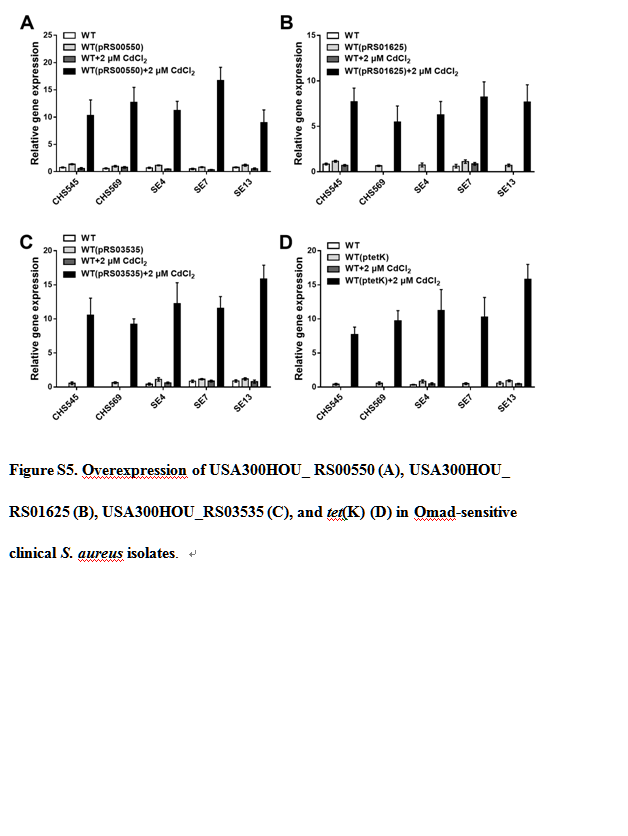

Supplement: Supplementary file 7 [file Image_5.PNG]
